# Efficient Oxygen Vacancy Defect Engineering for Enhancing Visible-Light Photocatalytic Performance over SnO_2−x_ Ultrafine Nanocrystals

**DOI:** 10.3390/nano12193342

**Published:** 2022-09-25

**Authors:** Tiekun Jia, Chenxi Sun, Nianfeng Shi, Dongsheng Yu, Fei Long, Ji Hu, Jilin Wang, Binbin Dong, Jili Li, Fang Fu, Shujing Hu, Joong Hee Lee

**Affiliations:** 1 Henan Province International Joint Laboratory of Materials for Solar Energy Conversion and Lithium Sodium Based Battery, Luoyang Institute of Science and Technology, Luoyang 471023, China; 2 School of Materials Science and Engineering, Guilin University of Technology, Guilin 541004, China; 3 Henan Province Engineering Research Center of Industrial Intelligent Vision, Luoyang Institute of Science and Technology, Luoyang 471023, China; 4 Department of Nano Convergence Engineering, Jeonbuk National University, Jeonju 54896, Korea

**Keywords:** hydrothermal synthesis, SnO_2−x_, oxygen vacancy, visible-light, photocatalytic degradation

## Abstract

Regardless of its good electron-transfer ability and chemical stability, pure Zn_2_SnO_4_ (ZSO) still has intrinsic deficiencies of a narrow spectral response region, poor absorption ability, and high photo-activated carrier recombination rate. Aiming to overcome the deficiencies above-mentioned, we designed a facile hydrothermal route for etching ZSO nanoparticles in a dilute acetic acid solution, through which efficient oxygen vacancy defect engineering was accomplished and SnO_2−x_ nanocrystals were obtained with an ultrafine particle size. In comparison with the untreated ZSO nanoparticles, the specific surface area of SnO_2−x_ nanocrystals was substantially enlarged, subsequently leading to the notable augmentation of active sites for the photo-degradation reaction. Aside from the above, it is worth noting that SnO_2−x_ nanocrystals were endowed with a broad spectral response, enhancing light absorption capacity and the photo-activated carrier transfer rate with the aid of oxygen vacancy defect engineering. Accordingly, SnO_2−x_ nanocrystals exhibited significantly enhanced photoactivity toward the degradation of the organic dye rhodamine B (RhB), which could be imputed to the synergistic effect of increasing active sites, intensified visible-light harvesting, and the separation rate of the photo-activated charge carrier caused by the oxygen vacancy defect engineering. In addition, these findings will inspire us to open up a novel pathway to design and prepare oxide compound photocatalysts modified by oxygen vacancy defects in pursuing excellent visible-light photoactivity.

## 1. Introduction

In the 21st century, human society has been entering into a new stage of rapid development, and the industrialization process has increasingly intensified, simultaneously accompanied by the two major serious problems of environment pollution and energy source shortage. Thus, how to reasonably address the two problems above-mentioned has become the focus of global concern. As is well-known, water pollution is one of the most important types of environment pollution, which originates from industrial discharge into the wastewater, containing pesticides, polychlorinated biphenyls, chlorophenols, dyes, and other organic pollutants [1,2,3,4,5,6]. Among these organic pollutants, dyes are considered as serious contributors to water pollution, because almost all textile industries excessively use different kinds of dyes, resulting in an excess of dyes being discharged into industrial waste water. Some of the dyes are hard to naturally decompose in water as their composite molecular structures can enable them to be more stable and resistant to biodegradation [7]. Therefore, it is extremely urgent to explore novel efficient methods to remove hazardous dyes.

In contaminated water without secondary pollution, among the physical, chemical, biological and acoustical methods of dye removal, advanced oxidation processes (AOPs) are reckoned as potentially effective strategies to remediate water pollution due to its main merits of easy control, ecological safety, and sufficient decomposition to different dyes. More interestingly, AOPs can also produce highly reactive peroxy and hydroxyl radicals to dominate the oxidation reaction of composite organic molecules [8,9,10,11,12,13,14,15]. Nowadays, the photodegradation process of dyes has become one of the most widely investigated AOPs, as it can covert solar energy into steady chemical energy and generate reactive species to decompose composite molecular structured substances [16,17,18]. Although the photodegradation of dyes has made great progress since the first discovery of photocatalytic hydrogen evolution over the TiO_2_ electrode [19], the photocatalytic efficiency toward the photodegradation of dyes is still limited to a narrow spectral response region, low solar-energy conversion efficiency, poor absorption ability, and high photo-activated carrier recombination rate of wide band-gap photo-catalysts. Therefore, developing novel catalysts with higher solar-energy conversion and photo-degradation efficiency to address the remediation of water pollution is the primary task for scientific researchers worldwide.

Oxygen defect engineering is a reliable and efficient approach to modulate the electronic structure of wide band-gap catalysts, resulting in narrowing the band-gap, enhancing the conductivity, and extending the light absorption range. Simultaneously, surface oxygen defects exert a critical influence on the efficient transfer and separation of photo-activated charge carriers [20,21,22,23]. Nowadays, oxygen defect engineering has been successfully utilized to modify TiO_2_, ZnO, SnO_2_, Bi_2_WO_6_, and other oxide compounds for the improvement of photodegradation efficiency [22,23,24,25,26,27,28,29,30,31,32,33,34,35,36,37,38]. For example, Feng et al. synthesized partially reduced TiO_2_ by using a facile one-step combustion method, and the reduced TiO_2_ exhibited improved hydrogen photocatalytic evolution under visible-light irradiation [24]. Li et al. previously designed and prepared a ternary structure of C@TiO_2_-TiO_2−x_ via a solvothermal route with enhanced photocatalytic performance [25]. With respect to ZnO, Xu et al. introduced a surface oxygen defect to narrow the band gap to achieve excellent solar-driven photodegradation toward tetracycline with long-term stability [30]. Moreover, Wang and his coauthors prepared ZnO with oxygen vacancy defects by annealing ZnO_2_ under different atmospheres, and their results showed that oxygen vacancy defects in crystals facilitated the photo-decomposition of 2,4- dichlorophenol in the presence of visible light illumination [31]. As for SnO_2−x_, some recent research work has also focused on the modification of oxygen vacancy. Dong and his coauthors adopted a molten-aluminum reduction route to achieve SnO_2−x_ nanoparticles with rich oxygen vacancy, and the SnO_2−x_ nanoparticles exhibited significantly improved visible-light absorption capacity and electrical property [32]. Anuchai et al. obtained oxygen-deficient SnO_2_ nanocrystals at a relatively low temperature, and confirmed the obtained samples with enhanced photodegradation performance [33]. Xu et al. designed a synproportionation reaction to achieve Sn^2+^ self-doped SnO_2−x_ with the improvement in the decomposition of methyl orange [34]. Chen et al. prepared SnO_2−x_/In_2_O_3−y_ composites with different vacuum levels, and investigated the relationship between the vacuum levels and the formation of oxygen vacancy [35]. All of the above findings on the SnO_2_ system with oxygen vacancy defects are impressive and inspiring; however, the present synthetic processes of SnO_2−x_ are still subject to multiple complicated steps, harsh conditions, and difficult control. Therefore, it still remains a great challenge to develop a facile approach for the large-scale synthesis of SnO_2−x_ ultrafine nanocrystals with oxygen vacancy defects for the significant improvement of photodegradation toward dyes.

As is well-known, pure Zn_2_SnO_4_ (ZSO), with a relatively wide bandgap approximate to SnO_2_, has good electron-transfer ability and chemical stability [39]. However, pure ZSO is still restricted by its intrinsic deficiencies of narrow spectral response region, poor absorption ability, and high photo-activated carrier recombination rate. Up to now, few studies have previously investigated the evolution of SnO_2−x_ ultrafine nanocrystals with oxygen vacancy defects from ZSO nanoparticles. Herein, we innovatively conceived a novel two-step hydrothermal route to achieve oxygen vacancy SnO_2−x_ nanocrystals. First, ZSO nanoparticles were hydrothermally obtained using SnCl_4_·5H_2_O and Zn(CH_3_COO)_2_·2H_2_O as starting materials. Second, efficient oxygen vacancy defect engineering was launched to achieve SnO_2−x_ nanocrystals by hydrothermally etching ZSO nanoparticles in a dilute acetic acid solution. In order to evaluate the effect of oxygen vacancies on the photoactivity, we carried out the photodegradation of rhodamine B (RhB) over different photocatalysts under visible-light irradiation. The results showed that SnO_2−x_ ultrafine nanocrystals with oxygen vacancy defects exhibited a higher photodecomposition efficiency compared to the pure ZSO nanoparticles. To our knowledge, this investigation is, for the first time, concerned with the preparation and photodecomposition behavior of SnO_2−x_ ultrafine nanocrystals originating from a hydrothermal etching process.

## 2. Materials and Methods

### 2.1. Materials

All of the chemicals used in this investigation including tin(IV) chloride penthydrate (SnCl_4_·5H_2_O), zinc acetate dehydrate (Zn(CH_3_COO)_2_·2H_2_O), sodium hydroxide (NaOH), and rhodamine B (RhB) were of analytical grade and purchased from Sinopharm Chemical Reagent Co. Ltd., Shanghai, China.

### 2.2. Synthesis of SnO_2−x_ Ultrafine Nanocrystals

According to our previous studies [40,41,42], first, ZSO nanoparticles were prepared through a facile hydrothermal route. The obtained white product was collected through centrifuging, washed with deionized water and ethanol, and then dried at 60 °C for 12 h.

Second, 0.5 g of ZSO nanoparticles was dissolved into a mixed solvent containing 72 mL deionized water and 8 mL acetic acid (HAC) under constant stirring. After being stirred for 30 min, the resulting suspension was poured into a 100 mL Teflon-lined autoclave. Then, the autoclave was heated to different temperatures and kept for 15 h. After naturally being cooled down to room temperature, the resulting precipitates were treated by centrifuging, washing, and drying. By varying different temperature (65 °C, 85 °C, 105 °C, 125 °C), a series of SnO_2−x_ ultrafine nanocrystals were obtained and respectively named as SnO_2−x_-65T, SnO_2−x_-85T, SnO_2−x_-105T, and SnO_2−x_-125T for convenience. For comparison, SnO_2_ nanoparticles were also synthesized via a hydrothermal route. The details for the synthetic process are described as follows. According to a previous study [43], a certain amount of SnCl_4_·5H_2_O and NaOH was dissolved into a mixed solvent containing 30 mL deionized water and 10 mL ethanol, respectively, then NaOH was added into the solution of SnCl_4_ until the pH reached 7. Then, the mixture was kept in a 100 mL Teflon-lined autoclave at 180 °C for 20 h. The final product was collected by centrifuging, washing, and drying.

### 2.3. Characterization

The X-ray diffraction patterns (XRD) of ZSO and SnO_2−x_ samples were obtained on an X-ray diffractometer (D8 Advance, Bruker, Billerica, MA, USA) with Cu-Kα irradiation (1.5406Å). The morphological observation of the as-prepared catalysts was accomplished using a transmission electron microscope (JEM-2100F, JEOL Ltd., Tokyo, Japan). To explore the surface composition and chemical status, X-ray photoelectron spectroscopy (XPS) measurements were achieved on an X-ray photoelectron spectrometer (XPS, ESCALAB 250Xi, Thermo Fisher Scientific cooperation), taking C1s (284.6 eV) as the criterion to calibrate the binding energies of other elements. Through the virtue of the nitrogen adsorption and desorption method, the specific surface area measurement was realized on a Tristar II 3020 sorption analyzer (Micromeritics, Norcross, GA, USA). Taking BaSO_4_ as the reflectance standard reference, UV–Vis diffuse reflectance spectra (DRS) of ZSO and SnO_2−x_ catalysts were recorded on a UV–Vis spectrophotometer (TU 1901, Puxi, Beijing, China). A LS55 fluorescence spectrometer (LS55, PE cooperation) was employed to measure the photoluminescence (PL) spectra of the ZSO and SnO_2−x_ samples with an exciting wavelength of 320 nm.

### 2.4. Photoelectrochemical Measurements

The electrochemical property was measured on a CHI660E electrochemical workstation comprising a standard three-electrode system, in which the working electrode, counter electrode, and reference electrode were made of as-obtained catalysts, Pt wire, and saturated Ag/AgCl, respectively. The detailed procedure on the electrochemical measurements was demonstrated in our previously reported study [44,45].

### 2.5. Photocatalytic Experiments

As reported in our previous research work [46], RhB photodecomposition experiments were carried out in a home-made device. In each run, we took RhB aqueous solutions (60 mL, 1.0 × 10^−5^ mol L^−1^) combined with 100 mg of catalysts. After being irradiated for a certain time, the absorbance intensities at the characteristic peak (554 nm) were recorded on a UV–Vis spectrophotometer to reflect the concentration variation of the reacted solution.

## 3. Results

### 3.1. Formation Mechanism of SnO_2−x_ Nanocrystals with Oxygen Vacancy

**Figure 1:**
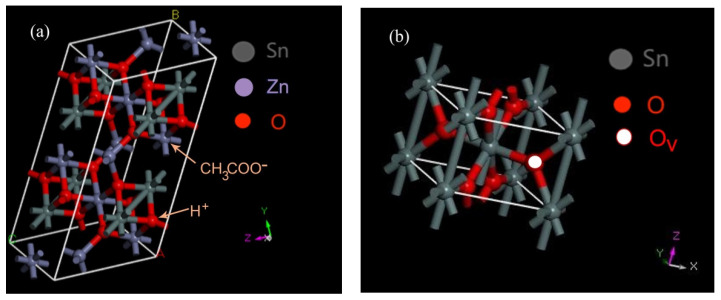
The formation schematic diagram of SnO_2−x_ nanocrystals in an acetic acid media under the hydrothermal condition. (**a**) ZSO structure; (**b**) SnO_2−x_ structure.

ZSO exhibits excellent chemical stability whether in an acid media or alkaline media under room temperature conditions. However, ZSO nanostructures are capable of being hydrothermally etched in alkaline media, which was verified in a previous study [46]. Similar to that of ZSO in alkaline media, the crystal structure of ZSO is likely to become unstable when undergoing a hydrothermal process in acid media. Thereby, a possible formation mechanism is proposed for the SnO_2−x_ nanocrystals as follows. With an increase in the hydrothermal temperature and pressure, the solubility of ZSO is progressively enhanced according to the thermodynamics under the hydrothermal condition. Simultaneously, a large number of CH_3_COO^−^ groups and hydrogen protons (H^+^) could be generated in the solution through acetic acid ionization, and the transfer rate of the hydrogen protons and CH_3_COO^−^ groups was significantly accelerated with an increase in the temperature upon the effect of thermal convection. When subjected to a certain concentration solution of HAC, Zn atoms located at crystal lattice of ZSO were capable of being attacked by groups of CH_3_COO^−^ from the solution, as depicted in Figure 1a. In the hydrothermal condition, Zn^2+^ can coordinate with CH_3_COO^−^ to generate a soluble component of Zn(CH_3_COO)_2_, which would subsequently enter into the solution through the solid–liquid interface between ZSO and HAC. At the same time, O atoms located at the crystal lattice of ZSO are also capable of being attacked by hydrogen protons in a weak acid hydrothermal environment, resulting in the formation of H_2_O. Thereafter, the crystal structure of ZSO is about to gradually dissociate and form a novel crystal structure under the constant etching of CH_3_COO^−^ groups and hydrogen protons. However, hydrogen protons are more active and provided with more rapid transfer rate in the hydrothermal etching process compared with the CH_3_COO^−^ groups. The factors above-mentioned could lead to the fact that the ratio of O atoms to Zn atoms stripped from the crystal lattice of ZSO is larger than 1:1. Thus, Sn atoms with partial O atoms remain therein, although the integrity of the ZSO crystal structure is destroyed. After experiencing the dissolution–recrystallization and in-situ growth process, non-stoichiometry SnO_2−x_ is about to form in a subsequent step, as depicted in Figure 1b.

### 3.2. Surface Composition and Defect Analysis

**Figure 2:**
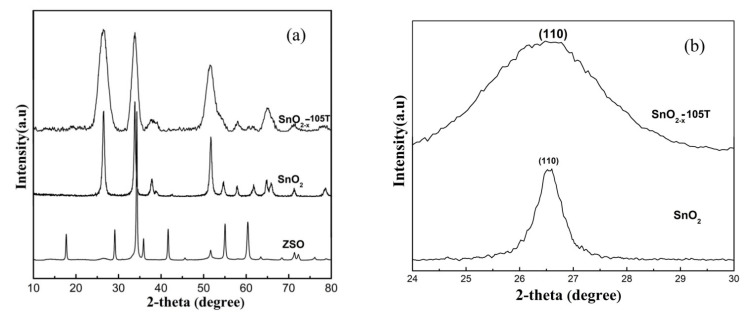
(**a**) XRD patterns of the ZSO, SnO_2_, and SnO_2−x_-105T samples; (**b**) partially enlarged diffraction peak of (110) plane of the SnO_2_ and SnO_2−x_-105T samples.

X-ray diffraction patterns were recorded to examine the variation of the crystal phase before and after undergoing an acid-assisted hydrothermal process. The XRD results are presented in Figure 2. For comparison, we also prepared pure SnO_2_ via a simple hydrothermal route, and its XRD pattern is presented in Figure 2. As clearly seen from Figure 2a, all diffraction peaks for the ZSO nanoparticles were relatively sharp and narrow, and they perfectly coincided with those of the hexagonal phase Zn_2_SnO_4_ (JCPDS No. 74-2184). After undergoing an acid-assisted hydrothermal process, obvious changes occurred in the diffraction patterns of the measured samples. The XRD diffraction patterns of SnO_2−x_-105T were almost in agreement with those of the tetragonal phase SnO_2_ (JCPDS No. 41-1445). Compared with those of ZSO and SnO_2_, the diffraction peaks became broader and weaker, suggesting that SnO_2−x_-105T might have a much smaller size, originating from the special acid-assisted hydrothermal process. Moreover, as is clearly seen from the (110) crystal plane in Figure 2b, the diffraction peak of SnO_2−x_-105T exhibited a slight shift in comparison with that of pure SnO_2_.

**Figure 3:**
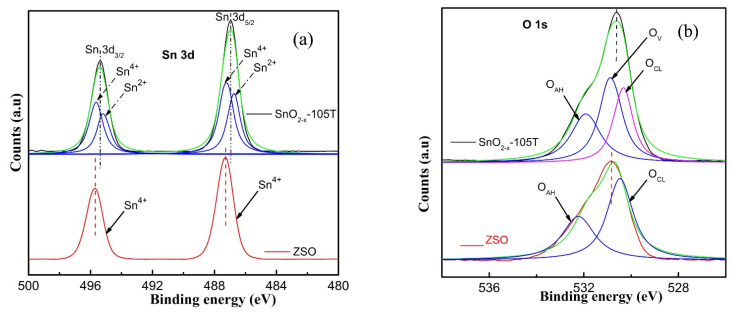
High resolution XPS spectra of the ZSO and SnO_2−x_-105T sample, (**a**) Sn 3d; (**b**) O 1s.

XPS measurements were executed to probe the surface composition and gain insights into the oxygen vacancy of the SnO_2−x_-105T sample. Figure 3 presents the high resolution XPS spectra of the Sn 3d and O1s of ZSO and SnO_2−x_-105T samples. It is very clear from Figure 3 that the SnO_2−x_-105T sample only consisted of Sn and O elements, coinciding with the results of the XRD measurements. Specifically, from Figure 3a, the Sn 3d orbital peaks for ZSO displayed two binding energies positioned at around 495.8 eV and 487.4 eV, respectively, corresponding to the Sn 3d_5/2_ and Sn 3d_3/2_ orbits. Obviously, Sn 3d_5/2_ and Sn 3d_3/2_ peaks of SnO_2−x_-105T (positioning at around 495.3 eV and 486.8 eV) exhibited a notable red-shift toward low energy direction compared with those of ZSO. According to the previously reported study [47], the binding energy (BE) of Sn 3d_5/2_ orbits for Sn^2+^ was in the region of 486.4 eV to 486.5 eV, while this value for Sn^4+^ ranged from 487.2 eV to 487.5eV. Thereby, it could reasonably be inferred that Sn^4+^, accompanied by Sn^2+^ ions, coexisted in the SnO_2−x_-105T sample because the BE of the Sn 3d peaks for SnO_2−x_-105T was slightly larger than those of Sn^2+^, but was a bit smaller than those of Sn^4+^ simultaneously. For this purpose, the Sn 3d_3/2_ spectrum could be deconvolved into two peaks centering at 495.8 eV and 495 eV, individually belonging to the Sn^4+^ and Sn^2+^ ions. As for the Sn 3d_5__/2_ spectrum, it can be analogously split into two characteristic peaks positioned at around 487.4 eV and 486.6 eV. Furthermore, the O 1s spectra were deeply examined to disclose the oxygen vacancy defect. As can clearly be seen in Figure 3b, the O 1s spectrum of ZSO was obviously different to that of SnO_2−x_-105T. Referring to previous work, the O1s orbital peak for SnO_2−x_-105T could be well-fitted with three characteristic peaks of the crystal lattice oxygen (O_CL_), oxygen vacancy (O_V_), and absorbed hydroxyl oxygen (O_AH_), individually located at around 530.5 eV, 531.1 eV, and 532.3 eV. As for ZSO, the O1s spectrum could be perfectly divided into the O_CL_ peak (530.5 eV) and O_AH_ peak (532.3 eV). The aforementioned result could provide strong proof to verify the existence of oxygen vacancy in SnO_2−x_-105T. In our work, it is rational to speculate that oxygen vacancy might originate from excess oxygen atoms stripped by hydrogen protons to form H_2_O, which leads to the variation in the electron density around Sn–O bond. Hence, the bonds of Sn^4+^–O–Sn^4+^ are partially substituted by the bonds of Sn^2+^–O–Sn^4+^ in order to compensate the oxygen vacancy to make the whole maintain charge neutrality. Based on the above demonstration, the XPS results are in good agreement with the formation mechanism of non-stoichiometry SnO_2−x_ nanocrystals.

### 3.3. Morphology and Surface Characteristics

**Figure 4:**
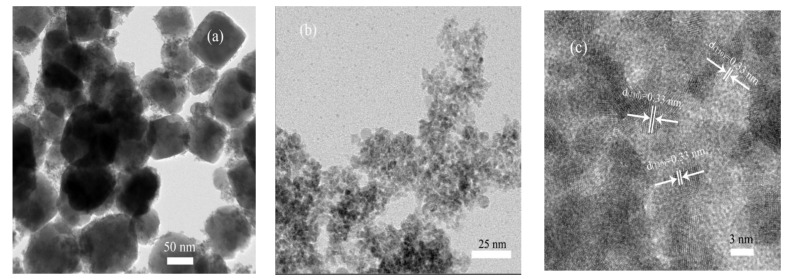
(**a**) TEM image of ZSO; (**b**) TEM image of SnO_2−x_-105T; (**c**) HRTEM image of SnO_2−x_-105T.

Figure 4 presents the TEM and HRTEM images of the ZSO and SnO_2−x_-105T samples. Through contrastive analysis from Figure 4a,b, an obvious difference was observed from the microstructure of the ZSO and SnO_2−x_-105T samples. Specifically, the ZSO sample was composed of numerous nanoparticles with relatively larger size, which was in good agreement with our previously reported study [40,41]. After undergoing the acid-assisted hydrothermal synthesis process, SnO_2−x_-105T exhibited a specially disordered mesostructure, and the crystal sizes significantly decreased, ranging from 5 nm to 7 nm. Such a special mesostructure of SnO_2−x_ can be attributed to the dissolution–recrystallization and in situ growth process. From Figure 4c, it is quite evident that the lattice fringes with a d-spacing of 0.33 nm corresponded to the (110) crystallographic planes of tin oxide. This result also revealed the good crystalline characteristic of ultra-fine SnO_2−x_ particles.

**Figure 5:**
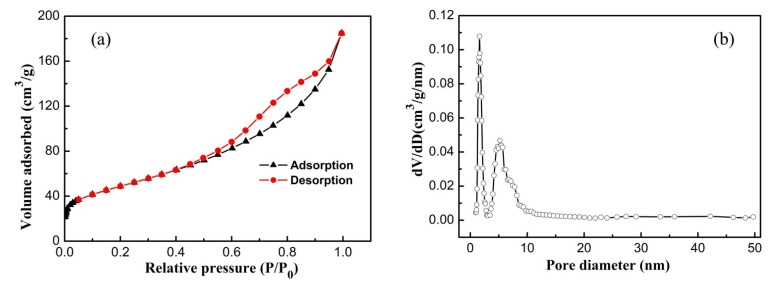
(**a**) The nitrogen adsorption–desorption isotherm and (**b**) the corresponding pore size and distribution curve of SnO_2−x_-105T.

The decrease in the nanocrystal size could enlarge the surface area of the SnO_2−x_-105T sample, which was mostly likely to intensify the absorption capacity and provide more active sites for the photodegradation reaction. In order to accurately analyze the surface area, the nitrogen sorption isotherm, together with the corresponding Barret–Joyner–Helenda (BJH) pore size distribution plot of the SnO_2−x_-105T sample, are presented in Figure 5. This is very distinct from Figure 5a, where the isotherm belongs to a type IV isotherm accompanied by a typical H3 loop according to IUPAC classification, indicating the mesoporous structure feature of the SnO_2−x_-105T sample. Abiding by the Brunauer–Emmett–Teller (BET) method, the specific surface area for the SnO_2−x_-105T sample was determined to be about 174.3 m^2^/g. This value was two times higher than that of ZSO (70.5 m^2^/g), which was previously reported in our work [40]. Thus, the specific surface area of the SnO_2−x_ nanocrystals was substantially enlarged. From Figure 5b, it is clear that two prominent peaks centered at about 1.6 nm and 5.2 nm were the main pore size distribution. Based on the analysis of microstructural observation and nitrogen sorption measurement, the formation of these tiny pores above-mentioned could probably be imputed to the stacking of ultra-fine SnO_2−x_ nanocrystals.

### 3.4. Optical Absorption and Band Energy Analysis

**Figure 6:**
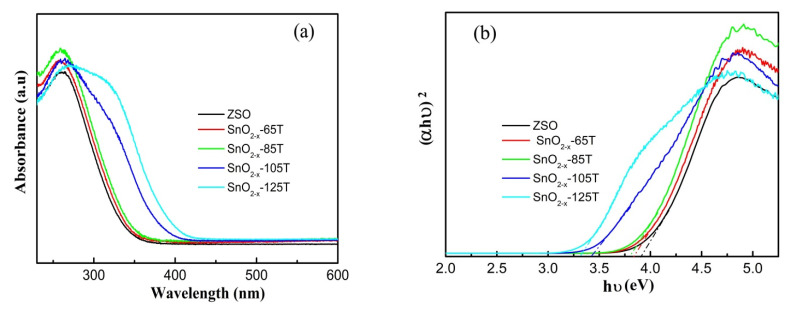
(**a**) UV–Vis absorbance spectra of the ZSO and SnO_2−x_ samples, (**b**) the derived plots of (*αhυ*)^2^ versus *hυ* from the absorption spectrum for ZSO and SnO_2−x_ samples.

As is evident from previous studies [26,29,30,31,33,34,35], the construction of oxygen vacancy engineering could broaden the spectral response and enhance the visible-light absorption capacity. In order to evaluate the light absorption property of the SnO_2−x_ samples, the UV–Vis diffraction reflectance spectra (DRS) of different catalysts are presented in Figure 6. A significant difference can be noticed between the ZSO and SnO_2−x_ samples in the DRS curves from Figure 6a. Specifically, ZSO only exhibited a strong absorption capacity in a narrow UV light region due to its wide band-gap (3.91 eV from Figure 6b). As for the SnO_2−x_ samples, the absorption edge displayed a stepwise redshift toward the visible-light region with the increase in the hydrothermal temperature compared with that of ZSO. Moreover, the extended tail located at the visible-light region in the absorbance curve was ascribed to the electron transfer associated with the oxygen vacancy-induced energy level near the valence band edge [48,49,50,51]. To better discuss the effect of the etching temperature on the band-gap energy (Eg) of the SnO_2−x_ samples, the plots of photon energy (hν) with respect to (αhν)^2^ are presented in Figure 6b. As seen from Tauc plots, the Eg value was remarkably influenced with the increase in the etching temperature. Based on the results of the Tauc plots, the Eg values for SnO_2−x_ samples were respectively determined to be about 3.3, 3.41, 3.79, and 3.84 eV, which were smaller than the reported value (3.95 eV) of the SnO_2_ nanoparticles [34]. However, it should be clearly noted that the Eg values for the SnO_2−x_ samples were a little larger than those reported in previous work [33], mainly originating from the fine sizes of the nanoparticles. Based on the above analysis, the result of the UV–Vis DRS, combined with the XPS result, could favor the presence of oxygen vacancy defects and demonstrate the effect on the band-gap energy and energy level.

### 3.5. Evaluation of Photo-Degradation Performance

**Figure 7:**
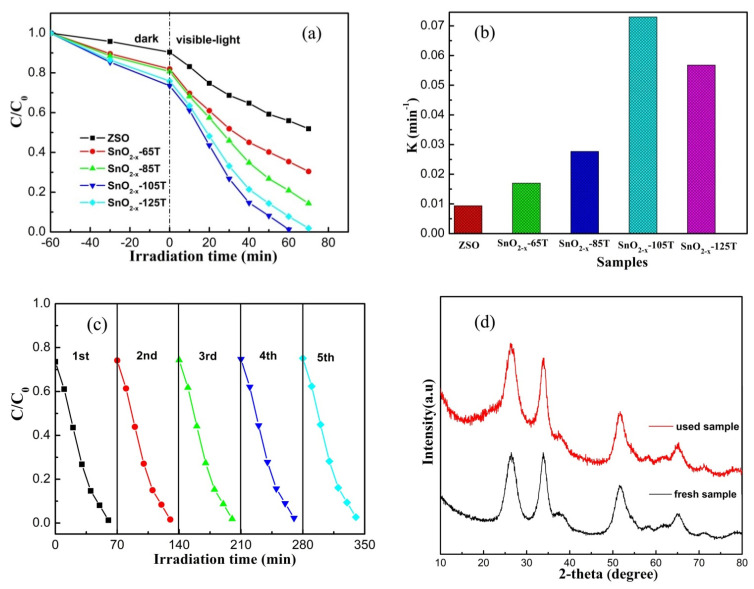
(**a**) The RhB photodegradation curves over different catalysts; (**b**) the corresponding apparent reaction rate constant; (**c**) cycling performance of the photodegradation of RhB solution over the SnO_2−x_-105T sample; (**d**) XRD pattern of SnO_2−x_-105T before and after use.

As one of the most important basic/cationic dyes, RhB is widely applied in the fields of textile, leather, printing paper, and pharmaceutics, which often result in the contamination of water sources due to its excess use. Due to its universal application and long-term stability, RhB was determined as the model pollutant in our work. Before visible-light illumination, RhB solution was placed in the dark for 1 h to make the reaction system reach the adsorption–desorption equilibrium. Figure 7a shows the RhB photo-decomposition plots over different catalysts. ZSO displayed inferior photodecomposition efficiency, and only 49% of the RhB molecules were decomposed upon 70 min of visible-light irradiation, probably originating from its wide band-gap and undesirable absorption property. Compared with ZSO, a larger amount of RhB molecules was absorbed on the surface of the SnO_2−x_ catalysts, suggesting their excellent absorption property. Simultaneously, a continuous enhancement in the photodecomposition efficiency of the SnO_2−x_ catalysts can clearly be seen in Figure 7a. Based on the BET results, combined with XPS and UV–Vis analysis, a larger BET area endowed SnO_2−x_ catalysts with excellent absorption capacity, and provided more active sites for the removal reaction of RhB molecules. Thus, the mesostructure, together with the energy level near valence band induced by the oxygen vacancy, mainly contributed to the substantial improvement in the removal rate of RhB molecules. Among the SnO_2−x_ catalysts, SnO_2−x_-105T displayed the highest removal efficiency, and about 98.75% of RhB molecules could be completely removed within 60 min. Given that the photodegradation reaction of RhB could adhere to the pseudo-first order reaction kinetics, the diagram for kinetic constant (k) values over different catalysts is depicted in Figure 7b. Correspondingly, the k value of the SnO_2−x_-105T sample was the largest among the tested catalysts, reaching 0.073 min^−1^, which was approximately seven times larger than that of ZSO. For this purpose, the SnO_2−x_-105T sample was determined as the representative catalyst in the subsequent measurements. Taking SnO_2−x_-105T as the model catalyst, cycling experiments were carried out to testify the usability and stability. After five successive cycling runs, no evident loss of the removal rate of RhB was detectable, revealing the high stability of the SnO_2−x_-105T sample. In comparison, the crystal phase of the used catalysts was in good accordance with that of the fresh catalyst, as seen in the XRD patterns from Figure 7d. This result further demonstrates the excellent usability and stability of the SnO_2−x_-105T sample.

### 3.6. Possible Enhancement Mechanism

Based on the afore-mentioned analysis, the intensification of adsorption capacity and the augment of BET area exerted a significantly positive effect on the enhancement of photodegradation toward the RhB solution. Aside from the above, oxygen vacancy defects can modulate the electronic structure, and induce the generation of novel energy levels near the valence band position for the n-type semiconductor catalyst, which would further influence the recombination rate as well as the separation rate of photo-activated electron-hole pairs. In order to ascertain this issue, PL measurement was carried out on the ZSO and SnO_2−x_ samples. Among all of the tested catalysts, ZSO possessed the strongest emission peak, fitting well with the worst removal rate of RhB. After the oxygen vacancy defects were introduced, the PL intensity of the SnO_2−x_ samples significantly decreased with an increase in the hydrothermal temperature. Specifically, the SnO_2−x_-105T sample had the weakest PL intensity, whereas the PL intensity, in contrast, increased when the etching temperature was beyond 105 °C. It is reasonably inferred that a further increase in the etching temperature could induce the generation of more oxygen vacancy defects, which could act as the recombination center of photoactivated charge carriers. Thus, SnO_2−x_-125T had a stronger PL intensity than SnO_2−x_-105T. This variation trend in the PL spectra for different catalysts was in good agreement with that of the photodegradation performance.

**Figure 8:**
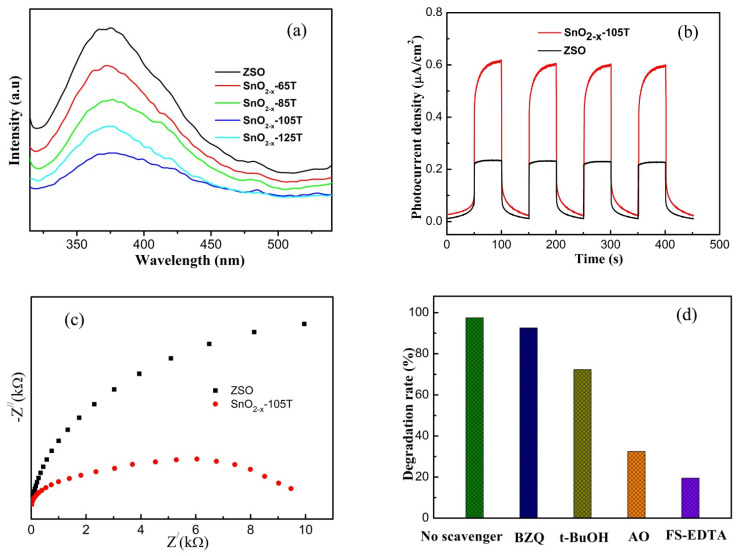
(**a**) PL spectra of the ZSO and SnO_2−x_ samples; (**b**) transient photocurrents and (**c**) electrochemical impedance spectra ZSO and SnO_2−x_-105T of electrodes under visible light irradiation; (**d**) the effect of various scavengers on the visible light photocatalytic performance of the SnO_2−x_-105T sample.

In order to further the investigation of the photo-activated electron transfer process, electrochemical measurements were executed, and the results are shown in Figure 8b,c. Generally, a larger photocurrent is tightly associated with a lower recombination rate of charge carriers, and more effective photoelectron transfer. In comparison, it is clear from Figure 8b that the photocurrent of the SnO_2−x_-105T sample was about three times higher than that of ZSO, demonstrating that the SnO_2−x_-105T sample had a much higher separation and transfer rate of photo-activated charge carriers. Additionally, the SnO_2−x_-105T sample displayed a substantial enhancement in photocurrents, equally confirming the influence of oxygen vacancy defects on band gap narrowing. As is well-known, a smaller arc radius in an EIS Nyquist plot signifies a lower electrochemical charge migration resistance at the interface. Obviously, the SnO_2−x_-105T sample had a lower electrochemical charge resistance, because the semicircle of its EIS Nyquist plot was smaller than that of ZSO, as observed in Figure 8c. Likewise, the result of the EIS measurement revealed the better capacity of the photoelectron migration of SnO_2−x_-105T, which agreed well with the photodecomposition test results.

To determine which active species were dominant in the photo-degradation of RhB over the SnO_2−x_ catalyst, radical trapping experiments were considered and performed in our work. Benzoquinone (BZQ 2 mmol L^−1^), tert-butyl-alcohol (*t*-BuOH 2 mmol L^−1^), ammonium oxalate (AO 2 mmol L^−1^), and FeSO_4_-EDTA (FS-EDTA, 0.2 mmol L^−1^) were taken as scavengers to capture·O_2_^−^, OH, h^+^, and H_2_O_2_, respectively, and the results are depicted in Figure 8d. Summarily, the restriction effect on the removal rate with the addition of four scavengers adhered to the order of FS-EDTA > AO > *t*-BuOH > BZQ. Clearly, the removal rate of RhB was remarkably suppressed upon exposure to FS-EDTA or AO, demonstrating that the contribution of h^+^ and H_2_O_2_ was primary radicals in the photo-decomposition process. Most notably, the RhB removal rate dwindled extraordinarily and significantly from approximately 98% to 20% due to the addition of FS-EDTA, demonstrating that the H_2_O_2_ radicals were the critical species to dominate the oxidation reaction system of the RhB and SnO_2−x_ catalyst.

**Figure 9:**
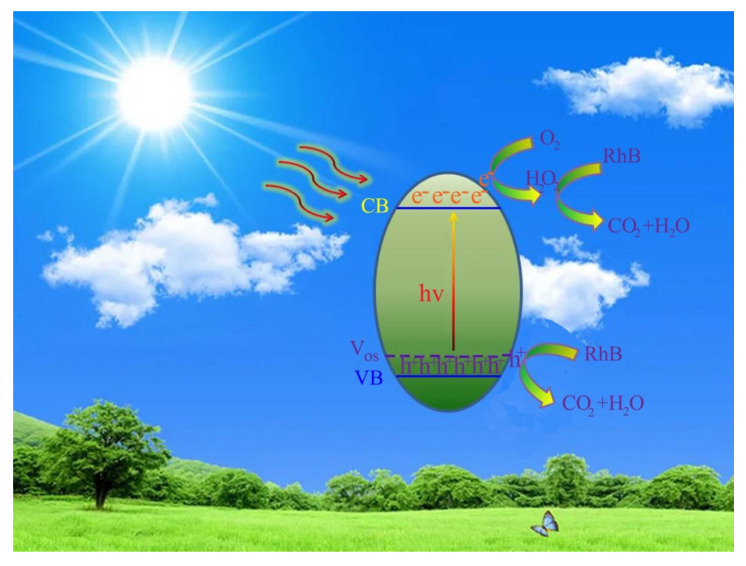
A schematic diagram of the proposed photodegradation mechanism over the SnO_2−x_ system under visible-light illumination.

In consultation with previously reported work [52], the conduction band (CB) edge potential for SnO_2_ was approximate to 0.05 eV. Thus, the valence band (VB) edge potential of SnO_2_ could be determined by the virtue of the equation of *E*_VB_ = *E*_CB_ + *E*_g_ (*E*_CB_ and E_VB_ represent CB and VB edge potential, respectively, while *E*_g_ refers to the band-gap). According to the demonstration above, a possible enhancement mechanism for the photodecomposition behavior is schematically presented in Figure 9. Herein, the novel energy level induced by oxygen vacancy defects is defined as “Vos”. Upon visible-light illumination, the electrons from the Vos position are capable of being activated, and transfer to the CB position. Note that the accumulated photo-activated electrons cannot reduce O_2_ to generate active radicals of ·O_2_^−^ because the CB potential of SnO_2−x_ was more positive than the potential of E^0^ ((O_2_/O_2_^−^) −0.33 eV vs. normal hydrogen electrode (NHE)) [53,54]. However, the CB potential of SnO_2−x_ was negative enough to make photoelectrons react with oxygen and generate the active radicals of H_2_O_2_ (O_2_/H_2_O_2_, 0.695 eV), which is mainly responsible for the photo-oxidation reaction of RhB. Simultaneously, the remaining photo-activated holes in Vos can directly oxidize RhB molecules to form CO_2_ and H_2_O. Therefore, the oxygen vacancy-induced energy level can act as an electron trap, further facilitating the generation and efficient transfer of photoelectrons, and finally produce more active species to significantly enhance the photo-activity toward the removal of RhB.

## 4. Conclusions

By controlling the ratio of O atoms to Zn atoms stripped from the crystal lattice of ZSO, we designed a novel two-step hydrothermal route to accomplish oxygen vacancy defect engineering. SnO_2−x_ ultrafine nanocrystals with oxygen vacancy defects were obtained after experiencing the dissolution–recrystallization and in situ growth process. Oxygen vacancy defects can adjust the electronic structure and induce the generation of the novel energy level near the valence band position. Aside from the augmentation of active sites and the improvement in the visible-light absorption capacity, the introduction of oxygen vacancy defects can also facilitate the generation and efficient transfer of photoelectrons under visible-light irradiation. Therefore, the synergistic effects of increasing active sites, intensified visible-light harvesting, and the photo-activated charge carrier’ separation rate could mainly contribute to the significant enhancement in the photodecomposition of RhB solution. This research will provide some enlightenment for the design and fabrication of a novel oxide compound semiconductor with oxygen vacancies toward the efficient and tunable organic pollutant degradation in waste water.

## Data Availability

The data presented in this study are available in this article.

## References

[1] Lianos P. (2011). Production of electricity and hydrogen by photocatalytic degradation of organic wastes in a photoelectrochemical cell: The concept of the photofuel cell: A review of a re-emerging research field. J. Hazard. Mater..

[2] Tang J., Zou Z., Ye J. (2004). Efficient photocatalytic decomposition of organic contaminants over CaBi_2_O_4_ under visible-light irradiation. Angew. Chem. Int. Ed..

[3] Reddy V.L.P., Kim K.A. (2015). Review of photochemical approaches for the treatment of a wide range of pesticides. J. Hazard. Mater..

[4] Shinde S.S., Bhosale C.H., Rajpure K.Y. (2014). Photodegradation of organic pollutants using N-titanium oxide catalyst. J. Photochem. Photobiol. B.

[5] Jo W., Tayade R.J. (2014). New generation energy-efficient light source for photocatalysis: LEDs for environmental applications. Ind. Eng. Chem. Res..

[6] Jo W., Tayade R.J. (2014). Recent developments in photocatalytic dye degradation upon irradiation with energy-efficient light emitting diodes. Chin. J. Catal..

[7] Mckay G. (1979). Waste colour removal from textile effluents. Am. Dyest. Rep..

[8] Hage R., Lienke A. (2006). Applications of transition-metal catalysts to textile and wood-pulp bleaching. Angew. Chem. Int. Ed..

[9] Hong Q., Hardcastle J.L., Mckeown R.A.J., Marken F., Compton R.G. (1999). The 20 kHz sonochemical degradation of trace cyanide and dye stuffs in aqueous media. New J. Chem..

[10] Wang S.A. (2008). Comparative study of fenton and fenton-like reaction kinetics in decolourisation of waste water. Dyes Pigments.

[11] Ma T.Y., Qiao S.Z. (2014). Acid—Base bifunctional periodic mesoporous metal phosphonates for synergistically and heterogeneously catalyzing CO_2_ conversion. ACS Catal..

[12] Shi B., Li G., Wang D., Feng C., Tang H. (2007). Removal of direct dyes by coagulation: The performance of preformed polymeric aluminum species. J. Hazard. Mater..

[13] Maezawa A., Nakadoi H., Suzuki K., Furusawa T., Suzuki Y., Uchida S. (2007). Treatment of dye wastewater by using photo-catalytic oxidation with sonication. Ultrason. Sonochem..

[14] Zhao Y.Y., Deng N., Fan Z.H., Hu Z.T., Fan L., Zhou J., Huang X. (2022). On-site H_2_O_2_ electro-generation process combined with ultraviolet: A promising approach for odorous compounds purification in drinking water system. Chem. Eng. J..

[15] Rui J.C., Deng N., Zhao Y.Y., Tao C., Zhou J.Z., Zhao Z., Huang X. (2022). Activation of persulfate via Mn doped Mg/Al layered double hydroxide for effective degradation of organics: Insights from chemical and structural variability of catalyst. Chemosphere.

[16] Wang H., Zhang L., Chen Z., Hu J., Li S., Wang Z., Liu J., Wang X. (2014). Semiconductor heterojunction photocatalysts: Design, construction, and photocatalytic performances. Chem. Soc. Rev..

[17] Yu H., Jiang L., Wang H., Huang B., Yuan X., Huang J., Zhang J., Zeng G. (2019). Modulation of Bi_2_MoO_6_-based materials for photocatalytic water splitting and environmental application: A critical review. Small.

[18] Li X., Xie J., Jiang C.J., Yu J.G., Zhang P.Y. (2018). Review on design and evaluation of environmental photocatalysts. Front. Environ. Sci. Eng..

[19] Fujishima A., Honda K. (1972). Electrochemical photolysis of water at a semiconductor electrode. Nature.

[20] Hu W., Liu Y., Withers R.L., Frankcombe T.J., Norén L., Snashall A., Kitchin M., Smith P., Gong B., Chen H. (2013). Electron-pinned defect-dipoles for high-performance colossal permittivity materials. Nat. Mater..

[21] Liu S.D., Kang L., Zhang J., Jun S.C., Yamauchi Y. (2021). Carbonaceous anode materials for nonaqueous sodium- and potassium-ion hybrid capacitors. ACS Energy Lett..

[22] Ning J., Mu C., Guo X., Yang R., Jonathan R., Jiao W., Wu X., Jian X. (2022). Efficient defect engineering and in-situ carbon doping in ultra-fine TiO_2_ with enhanced visible-light-response photocatalytic performance. J. Alloys Compd..

[23] Yang Y., Yin L.C., Gong Y., Niu P., Wang J.Q., Gu L., Chen X., Liu G., Wang L., Cheng H.M. (2018). An unusual strong visible-light absorption band in red anatase TiO_2_ photocatalyst induced by atomic hydrogen-occupied oxygen vacancies. Adv. Mater..

[24] Zuo F., Wang L., Wu T., Zhang Z., Borchardt D., Feng P.Y. (2010). Self doped Ti^3+^ enhanced photocatalytic for hydrogen production under visible-light. J. Am. Chem. Soc..

[25] Li Y., Xue J., Shen Q., Jia S., Li Q., Li Y., Liu X., Jia H. (2021). Construction of a ternary spatial junction in yolk-shell nanoreactor for efficient photo-thermal catalytic hydrogen generation. Chem. Eng. J..

[26] Wu J., Qiao P., Li H., Xu Y., Yang W., Yang F., Lin K., Pan K., Zhou W. (2020). Engineering surface defects on two-dimensional ultrathin mesoporous anatase TiO_2_ nanosheets for efficient charge separation and exceptional solar-driven photocatalytic hydrogen evolution. J. Mater. Chem. C.

[27] Li L., Chen X.H., Wang L., Tao C.Y., Wu X.P., Du J., Liu Z.H. (2020). Synthesis of Ti^3+^ self-doped mesoporous TiO_2_ cube with enhanced visible-light photoactivity by a simple reduction method. J. Alloys Compd..

[28] Sun D., Chi D., Yang Z., Xing Z., Chen P., Li Z., Pan K., Zhou W. (2020). CdS quantum dots modified surface oxygen vacancy defect ZnO_1−x_-TiO_2−x_ solid solution sphere as Z-Scheme heterojunctions for efficient visible light-driven photothermal-photocatalytic performance. J. Alloys Compd..

[29] Gurylev V., Perng T.P. (2021). Defect engineering of ZnO: Review on oxygen and zinc vacancies. J. Eur. Ceram. Soc..

[30] Xu Y.C., Li H., Sun B., Qiao P., Ren L., Tian G., Jiang B., Pan K., Zhou W. (2020). Surface oxygen vacancy defect-promoted electron-hole separation for porous defective ZnO hexagonal plates and enhanced solar-driven photocatalytic performance. Chem. Eng. J..

[31] Wang J., Wang Z., Huang B., Ma Y., Liu Y., Qin X., Zhang X., Dai Y. (2012). Oxygen vacancy induced band-gap narrowing and enhanced visible light photocatalytic activity of ZnO. ACS Appl. Mater. Interfaces.

[32] Dong W., Xu J., Wang C., Lu Y., Liu X., Wang X., Yuan X., Wang Z., Lin T., Sui M. (2017). A robust and conductive black tin oxide nanostructure makes efficient lithium-Ion batteries possible. Adv. Mater..

[33] Anuchai S., Phanichpahant S., Tantraviwat D., Pluengphon P., Bovornratanaraks T., Incessungvorn B. (2018). Low temperature preparation of oxygen-deficient tin dioxide nanocrystals and a role of oxygen vacancy in photocatalytic activity improvement. J. Colloid Interface Sci..

[34] Fan C.M., Peng Y., Zhu Q., Lin L., Wang R., Xu A.W. (2013). Synproportionation reaction for the fabrication of Sn^2+^ self-doped SnO_2−x_ nanocrystals with tunable band structure and highly efficient visible light photocatalytic activity. J. Phy. Chem. C.

[35] Liu W.T., Wu B., Lai Y., Tai N., Perng T., Chen L.J. (2018). Enhancement of water splitting by controlling the amount of vacancies with varying vacuum level in the synthesis system of SnO_2−x_/In_2_O_3−y_ heterostructure as photocatalyst. Nano Energy.

[36] Zhou Z.H., Liu J., Long R., Li L., Guo L.J., Prezhdo O.V. (2017). Control of charge carriers trapping and relaxation in hematite by oxygen vacancy charge: Ab initio non-adiabatic molecular dynamics. J. Am. Chem. Soc..

[37] Wu J.X., Qiao P., Li H., Ren L., Xu Y., Tian G., Li M., Pan K., Zhou W. (2019). Surface-oxygen vacancy defect-promoted electron-hole separation of defective tungsten trioxide ultrathin nanosheets and their enhanced solar-driven photocatalytic performance. J. Colloid Interface Sci..

[38] Kong X.Y., Lee W.Q., Mohamed A.R., Chai S.P. (2019). Effective steering of charge flow through synergistic inducing oxygen vacancy defects and *p-n* heterojunctions in 2D/2D surface-engineered Bi_2_WO_6_/BiOI cascade: Towards superior photocatalytic CO_2_ reduction activity. Chem. Eng. J..

[39] Tan B., Toman E., Li Y.G., Wu Y.Y. (2007). Zinc stannate (Zn_2_SnO_4_) dye-sensitized solar cells. J. Am. Chem. Soc..

[40] Jia T.K., Fu F., Long F., Min Z.Y., Zhao J.W., Chen J., Li J.L. (2016). Synthesis, characterization and enhanced visible-light photocatalytic activity of Zn_2_SnO_4_/C nanocomposites with truncated octahedron morphology. Ceram. Int..

[41] Jia T.K., Liu M., Yu D.Y., Long F., Mo S., Deng Z., Wang W. (2018). A facile approach for the synthesis of Zn_2_SnO_4_/BiOBr nanocomposites with improved visible light photocatalytic performance. Nanomaterials.

[42] Jia T.K., Fu F., Li J., Wang W., Hu X. (2019). Constructing a novel Zn_2_SnO_4_/C/AgBr nanocomposite with extended spectral Response and improved photocatalytic performance. J. Alloys Compd..

[43] Tuan P.V., Hieu L.T., Nga L.Q., Dung N.D., Ha N.N., Khiem T.N. (2016). Hydrothermal synthesis and characteristic photoluminescence of Er-doped SnO_2_ nanoparticles. Physica B.

[44] Jia T.K., Fu F., Li J.L., Deng D., Long F., Yu D., Cui Q., Wang W.M. (2020). Rational construction of direct Z-scheme SnS-g-C_3_N_4_ hybrid photocatalyst for significant enhancement of visible-light photocatalyticactivity. Appl. Surf. Sci..

[45] Yu D., Jia T.K., Deng Z., Wei Q., Wang K., Chen L., Wang P., Cui J. (2022). One-dimensional P-doped graphitic carbon nitride tube: Facile synthesis, effect of doping concentration, and enhanced mechanism for photocatalytic hydrogen evolution. Nanomaterials.

[46] Jia T.K., An J.C., Yu D., Li J., Fu F., Wang K., Wang W. (2019). Continuously improved photocatalytic performanceof Zn_2_SnO_4_/SnO_2_/Cu_2_O composites by structural modulation and band alignment modification. Nanomaterials.

[47] Chetri P., Choudhury B., Choudhury A. (2014). Room temperature ferromagnetism in SnO_2_ nanoparticles: An experimental and density functional study. J. Mater. Chem. C.

[48] Xu Y., Zheng L., Yang C., Zheng W., Liu X., Zhang J. (2020). Oxygen vacancies enabled porous SnO_2_ thin films for highly sensitive detection of triethylamine at room temperature. ACS Appl. Mater. Interfaces.

[49] Song M., Wu Y., Zhao Y., Du C., Su Y. (2020). Structural insight on defect-rich tin oxide for smart band alignment engineering and tunable visible-light-driven hydrogen evolution. Inorg. Chem..

[50] Li P., Liu Y., Zhang Q., Zhao Z., Pullerits T., Zheng K., Zhou Y. (2016). Iodinated SnO_2_ quantum dots: A facile and efficient approach to increase solar absorption for visible-light photocatalysis. J. Phys. Chem. C.

[51] Wang H., Dou K., Teoh W.-Y., Zhan Y., Hung T.-F., Zhang F., Xu J., Zhang R., Rogach A.L. (2013). Engineering of facets, band structure, and gas-sensing properties of hierarchical Sn^2+^-doped SnO_2_ nanostructures. Adv. Funct. Mater..

[52] Li Y., Wu X., Ho W., Lv K., Li Q., Li M., Lee S.C. (2018). Graphene-induced formation of visible- light-responsive SnO_2_-Zn_2_SnO_4_ Z scheme photocatalyst with surface vacancy for the enhanced photoreactivity towards NO and acetone oxidation. Chem. Eng. J..

[53] Kim J., Lee C.W., Choi W. (2010). Platinized WO_3_ as an environmental photocatalyst that generates OH radicals under visible light. Environ. Sci. Technol..

[54] Wang X., Li S., Yu H., Yu J. (2011). In situ anion-exchange synthesis and photocatalytic activity of Ag_8_W_4_O_16_/AgCl-nanoparticle core–shell nanorods. J. Mol. Catal. A Chem..

